# Comparison of the Efficacy of Zoledronate and Denosumab in Patients with Acute Osteoporotic Vertebral Compression Fractures: A Randomized Controlled Trial

**DOI:** 10.3390/jcm13072040

**Published:** 2024-04-01

**Authors:** Seong Son, Michael Y. Oh, Byung-Rhae Yoo, Han-Byeol Park

**Affiliations:** 1 Department of Neurosurgery, Gil Medical Center, Gachon University College of Medicine, Incheon 21565, Republic of Korea; sonseong44@gmail.com (S.S.); byungryoo@gilhospital.com (B.-R.Y.);; 2 Department of Neurological Surgery, University of California, Irvine, CA 95064, USA

**Keywords:** compression fracture, denosumab, osteoporosis, treatment outcome, zoledronate

## Abstract

**Background**: The comparison of the efficacy of zoledronate and denosumab for treating osteoporosis is controversial, and few randomized controlled trials have compared these two drugs in practical patients with acute osteoporotic vertebral compression fractures (OVCFs). We conducted a randomized controlled study to compare the efficacy of zoledronate and denosumab in patients with acute OVCF, with a focus on the occurrence of new OVCF. **Methods**: We enrolled 206 subjects who had their first acute OVCF, without any previous history of osteoporosis medication. The patients were randomly assigned to receive either intravenous zoledronate once a year or subcutaneous denosumab twice a year. We investigated the OVCF recurrence, clinical outcome, bone mineral density (BMD), and bone turnover markers over 12 months. **Results**: The final cohort comprised 89 participants (mean age of 75.82 ± 9.34 years, including 74 women [83.15%]) in the zoledronate group and 86 patients (mean age of 75.53 ± 10.23 years, including 71 women [82.56%]) in the denosumab group. New OVCFs occurred in 8 patients (8.89%) in the zoledronate group and 11 patients (12.79%) in the denosumab group (odds ratio, 1.485 [95% confidence interval, 0.567–3.891], *p* = 0.419). No significant difference was observed in the survival analysis between the two groups (*p* = 0.407). The clinical outcome, including the visual analog scale score for pain and simple radiographic findings, did not differ between the two groups. The changes in BMD and bone turnover markers were also not significantly different between the two groups. Additionally, drug-related adverse events did not differ between the groups in terms of safety. **Conclusions**: The efficacy of zoledronate was comparable to that of denosumab in terms of the occurrence of new OVCFs, as well as of the overall clinical course in patients with their first acute OVCF. Notably, this study represents the first comparison of these two drugs in patients with acute OVCF. However, further research with large-scale and long-term follow-up is necessary.

## 1. Introduction

Osteoporotic vertebral compression fracture (OVCF), which is primarily caused by osteoporosis, is a common disease associated with disability in elderly patients [1]. The lifetime incidence of OVCF is 40–50% in women and 13–20% in men, and the prevalence of OVCF reaches over 1000 per 100,000 person years among patients aged over 70 years [2]. Pain resulting from OVCF causes discomfort in movement and is accordingly associated with various morbidities and mortality [3].

Moreover, after the first occurrence of OVCF, recurrence (occurrence of new OVCF) is not uncommon, with rates of 12.00–14.86% after one year and of 19.30–32.00% after two years [4,5]. Because this recurrence is associated with a repeated deterioration in quality of life and the burden of economic costs, identifying and preventing risk factors are important [6]. Osteoporosis is the most significant risk factor for OVCF recurrence, apart from aging, a low body mass index, steroid use, and vertebroplasty [4,5,7]. 

Zoledronic acid and denosumab, which are injected yearly and every 6 months, respectively, are both widely used as primary bone resorption inhibitors for osteoporosis, owing to their safety, effectiveness, and patient convenience [8]. Previous studies comparing their safety and effectiveness have confirmed that the two drugs are similar or that denosumab has slightly better effects, which remains controversial [9,10,11]. However, few randomized controlled trials (RTCs) have compared these two drugs for the preventive effect of OVCF recurrence and clinical outcomes in a practical patient with acute OVCF. 

In this RCT, we investigated the efficacy of zoledronate and denosumab in preventing new OVCFs and other clinical courses in patients with a first acute OVCF

## 2. Materials and Methods

### 2.1. Trial Design and Ethics 

This was a prospective, single center, randomized, open-label trial that included patients who had experienced an acute OVCF for the first time in their lives. 

The study was conducted in compliance with the 1964 Helsinki Declaration and its later amendments and was approved by our Institutional Review Board (GDIRB2019-358). This clinical trial was also registered in the Clinical Research Information Service of South Korea (KCT0005424).

### 2.2. Aims 

The aim of this clinical trial was to evaluate the non-inferiority of zoledronate compared to denosumab in preventing new OVCFs and to assess their clinical course in patients with an acute OVCF. 

The primary endpoint was the occurrence of new OVCFs during the 12-month follow-up. The secondary endpoints included pain relief; changes in plain X-ray findings, bone mineral density (BMD), and laboratory findings; and drug-related adverse events.

### 2.3. Sample Size 

The number of samples was calculated as follows:

$$N=\frac{{2(Z_{\alpha }+Z_{\beta })}^{2}\;P_{1}(1-P_{1})}{\delta^{2}}$$

Based on the results of previous clinical trials of zoledronate and denosumab [9,10,12,13,14,15], the probability of survival without recurrence (*P*_1_) was set to 0.88, and the non-inferiority margin (σ) of the main effect variable was set to 0.10. Using the above formula, with a significance level of 5% and a power of 80%, the sample size was calculated as 165 patients. Accounting for a dropout rate of 20%, we recruited a total of 206 patients. 

### 2.4. Subject Recruitment and Time Frame

Participants were recruited from an outpatient clinic specializing in spine disease and thoracolumbar OVCFs. Potential participants were screened to determine their eligibility according to the following inclusion and exclusion criteria, based on the National Health Insurance guidelines. 

The inclusion criteria were as follows: patients aged >50 years with osteoporosis (≤−2.5 of the worst T-score of two-level lumbar spine or hip measured by dual-energy X-ray absorptiometry [DXA]); single-level, acute non-pathologic OVCF in thoracolumbar spine within 2 weeks, confirmed by magnetic resonance imaging (MRI) for the first time in life; patients with no prior history of osteoporosis treatment except for calcium and/or Vitamin D; and those who voluntarily participated in this clinical trial and were willing to comply with the clinical trial protocol. 

The exclusion criteria were as follows: patients with contraindications to both drug administrations, including hypersensitivity, hypocalcemia, and renal impairment with creatinine clearance of <35 mL/min; and those who were considered unfit to participate in this clinical trial by the researchers.

The 206 registered patients were randomly assigned to receive either an intravenous administration of zoledronic acid (5 mg/100 mL) for >15 min at baseline (day 0) or a subcutaneous injection in the upper arm, upper thigh, or abdomen of denosumab (60 mg/1 mL) at baseline (day 0) and at 6 months ± 2 weeks. All patients also received supplemental oral daily calcium (1000–1500 mg) and vitamin D (400–1200 IU) [16]. 

**Figure 1:**
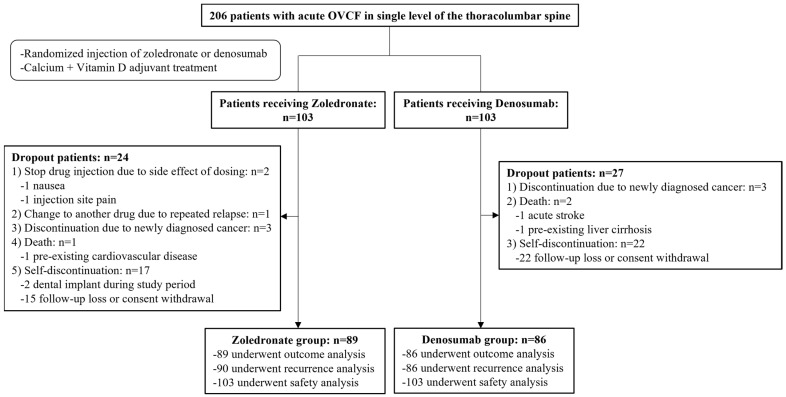
Diagram of participant recruitment and randomization.

The final follow-up period was 12 months, and the study was conducted from November 2019 to October 2021. Among the initially registered 206 participants, 175 were enrolled in the final cohort after excluding 31. OVCF recurrences were evaluated in 176 patients (90 in the zoledronate group and 86 in the denosumab group), and other outcomes were evaluated in 175 patients (89 in the zoledronate group and 86 in the denosumab group). Additionally, safety based on the occurrence of drug-related complications was evaluated in 206 patients who received a minimum dose of each drug (103 patients in each group) (Figure 1). 

### 2.5. Treatment Protocol for Acute OVCF 

The decision to perform conservative treatment, vertebroplasty/kyphoplasty, or fusion surgery was determined depending on the patient’s pain progression, the presence of neurological symptoms, and the patient’s decision. For conservative treatment, conventional analgesics, such as nonsteroidal anti-inflammatory drugs, tramadol, or opioids, were prescribed to control pain in an outpatient setting, and bed rest for 1–2 weeks was recommended. If pain relief was observed, conservative treatment was continued. Vertebroplasty or kyphoplasty was considered if severe pain or disability in daily life persisted, despite sufficient conservative treatment, and the patient agreed to surgery. Fusion surgery was performed when there was spinal canal compromise with neurological symptoms, such as progression of paralysis and bowel and bladder dysfunction. 

### 2.6. Detecting Occurrence of New OVCFs 

All patients were monitored for 1 year with regular clinic visits at 1 month ± 1 weeks, 6 months ± 2 weeks, and 12 months ± 4 weeks after drug administration for clinical surveys and examinations. Additionally, participants were instructed to visit the outpatient clinic immediately if they experienced any drastic changes in symptoms, such as exacerbation of pain or the development of neurological symptoms.

Plain X-rays of the thoracolumbar spine were taken at every visit to screen for new OVCFs or a recurrent OVCF at the same level. If any significant changes were identified on the radiography, the diagnosis was confirmed using an MRI.

### 2.7. Outcome Measurement of Overall Clinical Course

Quantitative parameter measurement and data collection were conducted after anonymous coding of the treatment group. 

Demographic data including age, sex, and fracture level were collected. Baseline characteristics related to the risk of osteoporotic fracture, including body mass index, smoking status, alcohol consumption status, rheumatoid arthritis, ankylosing spondylitis, steroid use, and a history of osteoporotic fracture, were recorded [17]. 

The degree of pain was assessed using a visual analog scale (VAS) ranging from 0 to 10 points on day 0, 1 month ± 1 weeks, 6 months ± 2 weeks, and 12 months ± 4 weeks after drug administration. 

**Figure 2:**
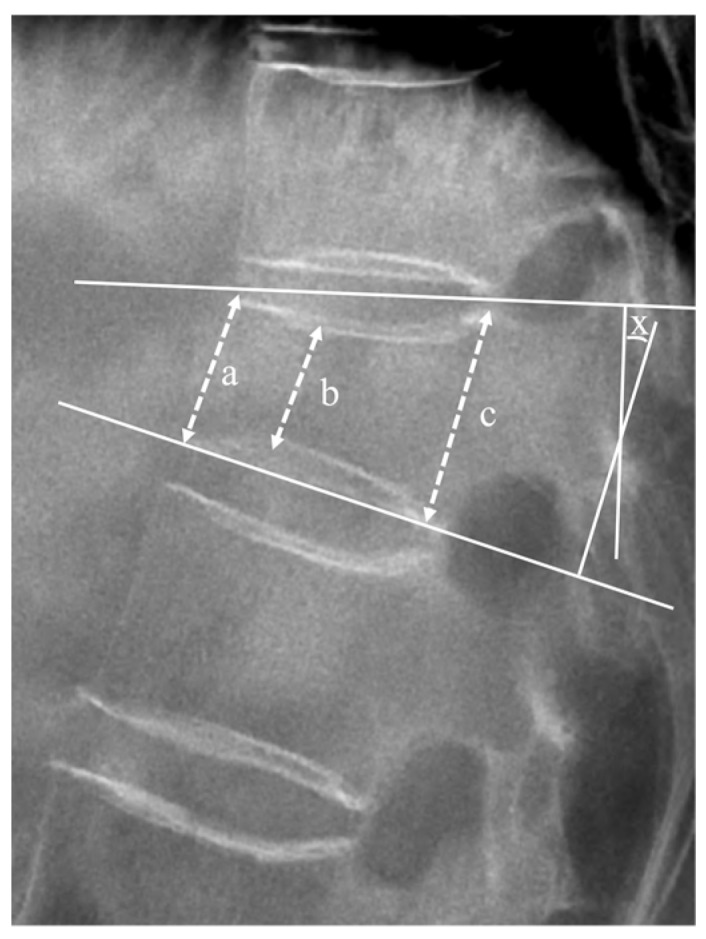
Lateral plain radiograph displaying measured factors. The mean height of the vertebral body was defined as the average of anterior margin (a), minimal height (b), and posterior margin (c) of the vertebral body, expressed as (a + b + c)/3. The vertebral height ratio (%) was calculated using the following equation: ($\frac{mean\;height\;of\;the\;index\;level}{\frac{mean\;height\;of\;the\;upper\;adjacent\;body\;+\;mean\;height\;of\;the\;lower\;adjacent\;body}{2}}$) × 100. The segmental angle (X) was measured as the angle formed by lines drawn along the superior and inferior plateaus of the vertebral body, where they intersect.

Radiological outcomes were evaluated using a plain X-ray before registration, and at the 6 months ± 2 weeks and 12 months ± 4 weeks follow-ups. The vertebral height ratio and segmental kyphotic angle (measured using Cobb’s method) on standing lateral radiographs in the neutral position were calculated to evaluate the progression of vertebral body collapse and deformity (Figure 2) [18,19]. 

The segmental kyphotic angle (x) was determined at the intersection of lines drawn at the superior plateau of the vertebral body and the inferior plateau of the vertebral body.

Laboratory tests were conducted for complete blood count, routine chemistry profile, calcium metabolism (including serum calcium, serum ionized calcium, 25-OH Vitamin D, 1,25[OH]_2_ Vitamin D3, thyroid hormone, and parathyroid hormone), and bone turnover markers (including alkaline phosphatase [ALP], osteocalcin, C-terminal telopeptide of type I collagen [CTx], and urine N-terminal telopeptide of type I collagen [NTx]) at day 0 and 12 months ± 4 weeks. 

To evaluate the BMD and T-score, a DXA of the lumbar spine and hip was performed before registration and at 12 months ± 4 weeks. The BMD and mean T-score of the lumbar spine and hip, except for the femur ward area, as well as changes in the BMD from the baseline, were analyzed. 

### 2.8. Drug-Related Adverse Events

Safety was assessed by the recording of all drug-related adverse events by the clinician. Adverse events were categorized according to the codes used in the Medical Dictionary for Regulatory Activities [20]. 

### 2.9. Statistical Analysis

Data management and statistical analyses were performed using SPSS software (version 27.0; IBM Corporation, Armonk, NY, USA). The results are expressed as a mean ± standard deviation, mean and corresponding 95% confidence interval (CI), or median with interquartile range (IQR), depending on the distribution of the data as assessed by the Kolmogorov–Smirnov test. Pearson’s chi-square test, Fisher’s exact test, the non-parametric Friedman test, non-parametric Mann–Whitney test, one-way analysis of variance, paired *t*-test, and independent *t*-test were used based on the characteristics of the factors. We used Kaplan–Meier survival analysis and log-rank tests to compare survival between the two groups. Statistical significance was defined as *p* < 0.05. 

## 3. Results

### 3.1. Demographic Data and Baseline Characteristics

The overall mean age was 75.67 ± 9.79, with 145 women (82.86%). The fracture levels were above T11 in 22 patients (12.57%), T11–T12 in 50 (28.57%), L1–L2 in 67 (38.29%), and L3–L5 in 36 (20.57%). The treatment strategy for acute OVCF comprised conservative treatment in 75 patients (42.86%), vertebroplasty/kyphoplasty in 97 patients (55.43%), and fusion in 3 patients (1.71%). 

Baseline characteristics, including age, sex ratio, body mass index, past medical history, fracture level, or treatment strategy, were not significantly different between the two groups, and all participants were Northeast Asian (Korean) (Table 1). 

### 3.2. Occurrence of New OVCFs

In the zoledronate group, new OVCFs occurred in 8 out of 90 patients (8.89%), with 7 patients at the adjacent level and 1 patient at the remote level. One patient suffered from repeated new OVCFs within 3 months and was dropped from the final cohort after receiving additional teriparatide therapy. In the denosumab group, new OVCFs occurred in 11 out of 86 patients (12.79%), with 9 patients at the adjacent level and 2 patients at the remote level. The odds ratio of the incidence of new OVCFs in the denosumab group compared with that in the zoledronate group was not significant at 1.485 (95% CI, 0.567–3.891, *p* = 0.419, Pearson’s chi-square test). 

**Figure 3:**
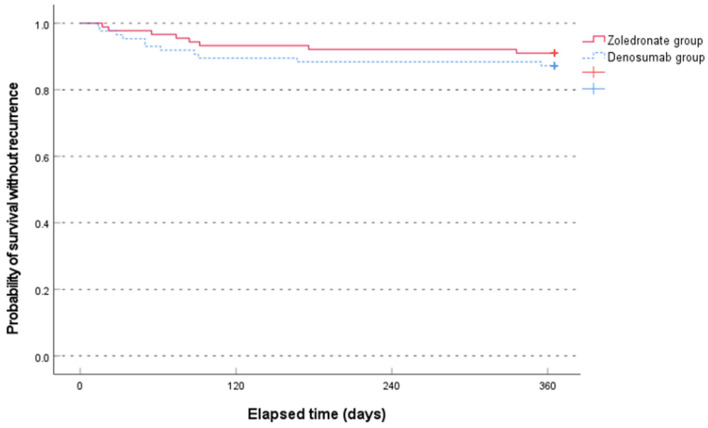
Comparison of the Kaplan–Meier survival analysis between the two groups for occurrence of new osteoporotic vertebral compression fractures for 1 year.

The median time to recurrence was not significantly different between the two groups (79.0 [range, 17.0–336.0] days in the zoledronate group and 50.0 [range, 14.0–355.0] days in the denosumab group, *p* = 0.481, non-parametric Mann–Whitney test). The Kaplan–Meier survival analysis showed no significant difference between the two groups (*p* = 0.407, log-rank test) (Figure 3).

### 3.3. Pain Relief

The median VAS score for back pain significantly improved longitudinally from baseline to 12 months in both groups (*p* < 0.001, non-parametric Friedman test). However, no significant intergroup differences in the VAS score for back pain at any of the follow-up visits were identified (Table 2).

### 3.4. Radiologic Outcomes 

The mean vertebral height ratio at the index levels significantly decreased longitudinally from baseline to 12 months in both groups (*p* < 0.001, paired *t*-test). However, there were no significant intergroup differences in the vertebral height ratio and segmental kyphotic angle at any of the follow-up visits (Table 3).

### 3.5. Laboratory Test

Laboratory values, including complete blood count, routine chemistry profile, and calcium metabolism, did not show significant differences between the two groups at baseline and at 1 year (Table 4). 

Overall bone turnover markers, including ALP, osteocalcin, CTx, and NTx, showed significant longitudinal decreases at 1 year compared to the baseline levels in both groups (*p* < 0.001, paired *t*-test). However, there were no significant differences in these markers between the two groups at any time point (Table 4). 

### 3.6. BMD and T-Score on DXA

In the zoledronate group, the mean BMD and mean T-score of the lumbar spine improved significantly (from 0.80 ± 0.12 to 0.87 ± 0.14 and from −2.99 ± 0.82 to −2.35 ± 1.18, *p* < 0.001, paired *t*-test). The mean BMD and mean T-score of the hip also improved significantly (from 0.64 ± 0.12 to 0.65 ± 0.11 and from −2.13 ±1.01 to −1.96 ± 0.96, *p* = 0.036 and *p* = 0.006, respectively, paired *t*-test).

In the denosumab group, the mean BMD and mean T-score of the lumbar spine improved significantly (from 0.81 ± 0.14 to 0.92 ± 0.19 and from −2.86 ± 1.21 to −1.98 ± 1.53, *p* < 0.001, paired *t*-test). The mean BMD of the hip also improved significantly from 0.61 ± 0.11 to 0.63 ± 0.13 (*p* = 0.049, paired *t*-test), while the mean T-score of the hip did not improve significantly (from −2.27 ± 0.92 to −2.15 ± 1.04, *p* = 0.096, paired *t*-test).

However, the mean values and the amount of change in the mean BMD and T-score were not significantly different between the two groups at each visit (Table 5). 

### 3.7. Drug-Related Adverse Events

In the zoledronate group, drug-related side effects were observed in 4 out of 103 patients (3.88%): 2 patients reported general arthralgia and myalgia within three days after the injection, 1 patient reported nausea during the drug injection, and 1 patient reported injection site pain/pyrexia during the injection. In the denosumab group, only 1 out of 103 patients (0.97%) reported drug-related side effects, specifically flatulence. Study discontinuation due to side effects occurred in the following two patients (1.94%) in the zoledronate group: one patient with nausea during the drug injection and one patient with injection site pain. However, no significant difference in the occurrence of drug-related adverse events was observed between the two groups (*p* = 0.370, Fisher’s exact test).

In terms of serious adverse events, there was one death due to pre-existing cardiovascular disease and three newly diagnosed cases of cancer during the study period in the zoledronate group. In the denosumab group, there were two deaths, including one due to acute stroke and one due to pre-existing liver cirrhosis, and three newly diagnosed cases of cancer during the study period. However, the investigator did not consider these serious adverse events to be directly related to the drugs (Table S1).

## 4. Discussion

### 4.1. Efficacy Regarding New OVCF Prevention and Clinical Outcomes 

The comparison between zoledronate and denosumab for preventing OVCFs has been controversial [21]. Although the two drugs were not directly compared, previous large-scale RCTs have revealed that the two drugs have a similar rate of relative risk reduction of new OVCFs compared to the control group, ranging from 68% to 70% [14,15,22,23,24]. Furthermore, according to a population-based study and meta-analyses with 2467 pairs of propensity score matching, the risk of OVCF after treatment was similar between the two drugs (HR, 1.21; 95% CI, 0.84–1.73) [9,11,25]. However, several previous studies have suggested that denosumab is more effective than zoledronate in preventing compression fractures in patients with metastasis or multiple myeloma [26,27,28]. 

According to this study, which directly compared the two drugs in patients with acute OVCF, the efficacy of preventing new OVCFs, and the clinical outcomes after the first OVCF, were similar between the two drugs. The occurrence of new OVCFs and comparative survival analysis did not differ significantly between the two groups at 12 months, although the recurrence rate was slightly higher in the denosumab group (12.79%) than in the zoledronate group (8.89%). Additionally, no significant differences in back pain relief and the progression of vertebral body collapse were observed between the two groups. 

### 4.2. Efficacy Regarding BMD Improvement and Bone Turnover Markers

The comparative results of the degree of BMD enhancement between zoledronate and denosumab are controversial. Several large-scale RCTs have reported that the change in BMD from baseline to 12 months was significantly greater in the denosumab group than in the zoledronate group at each skeletal site; the difference ranged from 0.6 to 2.1% [10,29]. Furthermore, a meta-analysis involving several RCTs has suggested that denosumab resulted in a higher BMD than zoledronate [8,22]. However, a retrospective study with a large cohort has reported that denosumab achieved similar increases to zoledronic acid in lumbar spine BMD, despite the more prominent reduction in bone turnover markers [9,13]. Moreover, some RCTs and a large population-based study have demonstrated that both zoledronate and denosumab increased BMD similarly at 12 months after the first administration in patients with postmenopausal osteoporosis, regardless of bisphosphonate pretreatment [30]. 

The present study, with a 12-month follow-up, found that changes in BMD and bone turnover markers did not significantly differ between the two groups. Although the improvement in mean BMD from baseline was slightly better in the denosumab group compared to the zoledronate group (6.38% versus 9.75% in the lumbar spine and 11.45% versus 14.47% in the hip), there was no statistical difference. However, in terms of the T-score, the zoledronate group showed a significant improvement in both lumbar spine and hip, while the denosumab group showed a significant improvement only in the lumbar spine. 

### 4.3. Safety Based on Adverse Events 

The safety of zoledronate and denosumab in terms of drug-related adverse events is controversial. Some authors have suggested that both drugs have comparable safety profile in terms of adverse event risk, including side effects, serious infections, and cardiovascular diseases [9,22]. However, several meta-analyses have reported denosumab as being safer than zoledronate [8,11,31]. 

According to this study, there was no statistically significant difference in drug-related adverse events between the two groups, although the incidence rate of side effects was higher in the zoledronate group than in the denosumab group (3.88% vs. 0.97%), and discontinuation of the study due to drug side effects was more common in the zoledronate group than in the denosumab group (2.91% vs. 0%). 

### 4.4. Limitation and Significance 

The present study has several limitations. First, the number of patients enrolled was not large. Nonetheless, considering the purpose of this study as a non-inferiority trial, it provides sufficient evidence to prove the relative efficacy of zoledronate compared to denosumab. Secondly, the follow-up period was relatively short. The results may vary with a follow-up period of >24 months. Third, the heterogeneity of treatment strategies for OVCF may have affected the outcomes as a confounding factor. For example, vertebroplasty/kyphoplasty can cause a bias in the BMD of the lumbar spine, pain relief, and radiologic outcomes after 12 months. Lastly, we did not control for other factors influencing bone turnover marker tests nor adjust the results for age and sex, potentially affecting the interpretation of these markers. 

However, to the best of our knowledge, RCTs on the efficacy between zoledronate and denosumab in terms of OVCF recurrence after an acute OVCF are rare. Accordingly, this study provides an important reference for clinicians considering the administration of osteoporosis drugs to prevent new fractures after the first acute OVCF. However, large-scale RCTs with a follow-up of >24 months are necessary to confirm the comparative efficacy of the two drugs. 

## 5. Conclusions

The overall short-term (12-month follow-up) results suggest that zoledronate is comparable to denosumab in terms of preventing new OVCFs, improving clinical outcomes, changing BMD and bone turnover markers, and preventing adverse events in patients with a first acute OVCF. However, due to the existence of controversial viewpoints, further investigation is necessary. 

## Figures and Tables

**Table 1:** Demographic data and baseline characteristics.

	Zoledronate Group (*n* = 89)	Denosumab Group (*n* = 86)	*p* Value
Age, years	75.82 ± 9.34	75.53 ± 10.23	0.844 ^†^
Sex ratio: male/female	15/74	15/71	0.918 ^‡^
Height, cm	155.31 ± 8.42	154.10 ± 8.87	0.379 ^†^
Weight, kg	55.31 ± 10.09	53.43 ± 8.40	0.199 ^†^
Body mass index, kg/cm^2^	22.86 ± 3.70	22.54 ± 3.57	0.587 ^†^
Smoking: yes/no	1/88	3/83	0.365 ^⸹^
Alcohol: yes/no	2/87	3/83	0.680 ^⸹^
Rheumatoid arthritis, n (%)	3 (3.37)	5 (5.81)	0.494 ^⸹^
Ankylosing spondylitis, n (%)	1 (1.12)	0	1.000 ^⸹^
Steroid use, n (%)	3 (3.37)	3 (3.49%)	1.000 ^⸹^
Previous osteoporotic fracture of another site, n (%)	1 (1.12)	3 (3.49%)	0.365 ^⸹^
Fracture level, n (%)			0.951 ^‡^
Above T11	12 (13.48)	10 (11.63)	
T11–T12	24 (26.97)	26 (30.23)	
L1–L2	35 (39.33)	32 (37.21)	
L3–L5	18 (20.22)	18 (20.93)	
Treatment of OVCF, n (%)			0.336 ^‡^
Conservative treatment	34 (39.53)	41 (47.67)	
Vertebroplasty or kyphoplasty	54 (60.67)	43 (50.00)	
Fusion	1 (1.12)	2 (2.33)	

OVCF, osteoporotic vertebral compression fracture. ^†^ Independent *t*-test, ^‡^ Pearson’s chi-square test, ^⸹^ Fisher’s exact test.

**Table 2:** Change in back pain based on visual analog scale.

	Zoledronate Group (*n* = 89)	Denosumab Group (*n* = 86)	*p* Value
VAS at baseline	7.00 (IQR, 5.00−8.00)	7.50 (IQR, 5.00−8.00)	0.869 ^†^
VAS at 1 month	4.00 (IQR, 3.00−6.75)	3.00 (IQR, 2.00−6.00)	0.818 ^†^
VAS at 6 months	3.00 (IQR, 1.00−5.00)	3.00 (IQR, 1.00−5.00)	0.476 ^†^
VAS at 1 year	2.50 (IQR, 1.00−3.00)	2.00 (IQR, 1.00−4.00)	0.693 ^†^

IQR, interquartile range; VAS, visual analog scale. ^†^ Non-parametric Mann–Whitney test.

**Table 3:** Radiologic outcomes.

	Zoledronate (*n* = 89)	Denosumab (*n* = 86)	*p* Value
Vertebral body height ratio, %			
Baseline	75.65 ± 12.44	74.12 ± 19.06	0.686 ^†^
6 months	72.12 ± 12.14	70.69 ± 20.51	0.438 ^†^
1 year	70.57 ± 8.63	69.85 ± 10.40	0.665 ^†^
Segmental kyphotic angle, °			
Baseline	8.58 ± 4.19	8.39 ± 4.47	0.907 ^†^
6 months	6.19 ± 3.35	6.20 ± 3.93	0.994 ^†^
1 year	5.59 ± 4.18	5.83 ± 4.84	0.380 ^†^

^†^ Independent *t*-test.

**Table 4:** Laboratory tests, including routine test and values related to bone metabolism.

	Zoledronate (*n* = 89)	Denosumab (*n* = 86)	*p* Value
Creatinine, mg/dL			
Baseline	0.73 ± 0.29	0.84 ± 0.34	0.124 ^†^
1 year	0.94 ± 0.37	0.90 ± 0.36	0.696 ^†^
Calcium, mg/dL			
Baseline	8.52 ± 0.57	8.41 ± 0.65	0.431 ^†^
1 year	8.85 ± 0.48	8.89 ± 0.61	0.832 ^†^
Ionized calcium, mmol/L			
Baseline	1.22 ± 0.06	1.20 ± 0.06	0.230 ^†^
1 year	1.23 ± 0.07	1.20 ± 0.05	0.052 ^†^
PTH, pg/mL			
Baseline	49.97 ± 23.71	63.49 ± 34.24	0.208 ^†^
1 year	48.91 ± 21.99	52.60 ± 25.90	0.487 ^†^
25(OH)Vitamin D, ng/mL			
Baseline	17.83 ± 7.98	22.85 ± 16.65	0.084 ^†^
1 year	25.44 ± 10.00	29.02 ± 16.02	0.237 ^†^
1,25(OH)_2_ Vitamin D3, pg/mL			
Baseline	35.75 ± 23.29	34.50 ± 23.55	0.811 ^†^
1 year	37.19 ± 21.72	39.12 ± 16.82	0.653 ^†^
ALP, IU/L			
Baseline	94.15 ± 35.93	93.23 ± 37.08	0.911 ^†^
1 year	70.28 ± 25.32	69.93 ± 25.66	0.951 ^†^
Osteocalcin, ng/mL			
Baseline	14.73 ± 7.73	15.72 ± 11.29	0.651 ^†^
1 year	12.00 ± 5.79	10.33 ± 7.52	0.264 ^†^
CTx, ng/mL			
Baseline	0.47 ± 0.29	0.44 ± 0.31	0.612 ^†^
1 year	0.24 ± 0.17	0.22 ± 0.20	0.638 ^†^
NTx, ng/mL			
Baseline	48.80 ± 32.37	40.39 ± 28.41	0.259 ^†^
1 year	26.23 ± 13.25	25.46 ± 15.44	0.817 ^†^

ALP, alkaline phosphatase; CTx, C-terminal telopeptide of type I collagen; NTx, urine N-terminal telopeptide of type I collagen; PTH, parathyroid hormone. ^†^ Independent *t*-test.

**Table 5:** Mean bone mineral density and T-score according to dual-energy X-ray absorptiometry.

	Zoledronate (*n* = 89)	Denosumab (*n* = 86)	Mean Difference	*p* Value
Mean BMD of the lumbar spine				
Baseline	0.80 ± 0.12	0.81 ± 0.14	0.01 (95%CI, −0.05–0.06)	0.787 ^†^
1 year	0.87 ± 0.14	0.92 ± 0.19	0.04 (95%CI, −0.02–0.11)	0.198 ^†^
Amount of change, %	6.38 (IQR, 0.33−17.05)	9.75 (IQR, 1.65−21.78)	6.21 (95%CI, −1.66–14.07)	0.174 ^‡^
Mean T-score of the lumbar spine				
Baseline	−2.99 ± 0.82	−2.86 ± 1.21	0.13 (95%CI, −0.30–0.56)	0.544 ^†^
1 year	−2.35 ± 1.18	−1.98 ± 1.53	0.38 (95%CI, −0.18–0.93)	0.183 ^†^
Mean BMD of the hip				
Baseline	0.64 ± 0.12	0.61 ± 0.11	0.02 (95%CI, −0.07–0.02)	0.325 ^†^
1 year	0.65 ± 0.11	0.63 ± 0.13	0.02 (95%CI, −0.07–0.02)	0.344 ^†^
Amount of change, %	11.45 (IQR, 0.38−16.74)	14.47 (IQR, 6.79−15.20)	3.98 (95%CI, −5.13–13.09)	0.121 ^‡^
Mean T-score of the hip				
Baseline	−2.13 ± 1.01	−2.27 ± 0.92	0.14 (95%CI, −0.26–0.53)	0.500 ^†^
1 year	−1.96 ± 0.96	−2.15 ± 1.04	0.20 (95%CI, −0.20–0.60)	0.337 ^†^

BMD, bone mineral density; IQR, interquartile range. ^†^ Independent *t*-test, ^‡^ non-parametric Mann–Whitney test.

## Data Availability

The data presented in this study are available from the corresponding author on receipt of reasonable request and are not publicly available.

## Supplementary Materials

The following supporting information can be downloaded at: https://www.mdpi.com/article/10.3390/jcm13072040/s1, Table S1: Serious adverse events
