# Supplementary material for: Comparison of the Efficacy of Zoledronate and Denosumab in Patients with Acute Osteoporotic Vertebral Compression Fractures: A Randomized Controlled Trial
Source: J Clin Med. 2024 Apr 1;13(7):2040. doi: 10.3390/jcm13072040 (PMC11012809; doi:10.3390/jcm13072040)
Supplement: Supplementary file 1 [file jcm-13-02040-s001.zip › jcm-2917662-supplementary.pdf]

Table S1. List of serious adverse events.

Group	Serious adverse event	Reason	Decision
Zoledronate	Expired due to cardia arrest	Pre-existed cardiovascular disease	No direct correlation with medication
Zoledronate	Colon cancer	Occurrence due to age and baseline risk factor	No direct correlation with medication
Zoledronate	Non-small cell lung cancer	Occurrence due to age	No direct correlation with medication
Zoledronate	Klatskin tumor	Occurrence due to age and family history	No direct correlation with medication
Denosumab	Expired due to complication of acute intracranial hemorrhage	Occurrence due to baseline risk factor	No direct correlation with medication
Denosumab	Expired due to complication of liver cirrhosis	Pre-existed liver cirrhosis	No direct correlation with medication
Denosumab	Multiple myeloma	Misdiagnosed as an osteoporotic compression fracture	No direct correlation with medication
Denosumab	Prostate cancer	Occurrence due to age and baseline risk factor	No direct correlation with medication
Denosumab	Pancreatic cancer	Occurrence due to age and baseline risk factor	No direct correlation with medication
